# Deep learning to estimate durable clinical benefit and prognosis from patients with non-small cell lung cancer treated with PD-1/PD-L1 blockade

**DOI:** 10.3389/fimmu.2022.960459

**Published:** 2022-11-07

**Authors:** Jie Peng, Jing Zhang, Dan Zou, Lushan Xiao, Honglian Ma, Xudong Zhang, Ya Li, Lijie Han, Baowen Xie

**Affiliations:** ^1^ Department of Medical Oncology, The Second Affiliated Hospital, Guizhou Medical University, Kaili, China; ^2^ Department of Radiology, Nanfang Hospital, Southern Medical University, Guangzhou, China; ^3^ Hepatology Unit and Department of Infectious Diseases, Nanfang Hospital, Southern Medical University, Guangzhou, China; ^4^ Department of Radiation Oncology, Cancer Hospital of the University of Chinese Academy of Sciences, Hanzhou, China; ^5^ The Second Department of Radiation Oncology, The First Affiliated Hospital of Zhengzhou University, Zhengzhou, China; ^6^ Department of Hematology, The First Affiliated Hospital of Zhengzhou University, Zhengzhou, China; ^7^ Yino Research, Shenzhen Yino Intelligence Technology Development Co., Ltd., Shenzhen, China

**Keywords:** deep learning, durable clinical benefit, non-small cell lung cancer, PD-1/PD-L1 blockade, prognosis

## Abstract

Different biomarkers based on genomics variants have been used to predict the response of patients treated with PD-1/programmed death receptor 1 ligand (PD-L1) blockade. We aimed to use deep-learning algorithm to estimate clinical benefit in patients with non-small-cell lung cancer (NSCLC) before immunotherapy. Peripheral blood samples or tumor tissues of 915 patients from three independent centers were profiled by whole-exome sequencing or next-generation sequencing. Based on convolutional neural network (CNN) and three conventional machine learning (cML) methods, we used multi-panels to train the models for predicting the durable clinical benefit (DCB) and combined them to develop a nomogram model for predicting prognosis. In the three cohorts, the CNN achieved the highest area under the curve of predicting DCB among cML, PD-L1 expression, and tumor mutational burden (area under the curve [AUC] = 0.965, 95% confidence interval [CI]: 0.949–0.978, *P<* 0.001; AUC =0.965, 95% CI: 0.940–0.989, *P*< 0.001; AUC = 0.959, 95% CI: 0.942–0.976, *P*< 0.001, respectively). Patients with CNN-high had longer progression-free survival (PFS) and overall survival (OS) than patients with CNN-low in the three cohorts. Subgroup analysis confirmed the efficient predictive ability of CNN. Combining three cML methods (CNN, SVM, and RF) yielded a robust comprehensive nomogram for predicting PFS and OS in the three cohorts (each *P*< 0.001). The proposed deep-learning method based on mutational genes revealed the potential value of clinical benefit prediction in patients with NSCLC and provides novel insights for combined machine learning in PD-1/PD-L1 blockade.

## Introduction

Immune checkpoint blockade (ICB) therapy has been proven to be successful as a treatment for non-small cell lung cancer (NSCLC) (1, 2), and different biomarkers, such as PD-L1 expression (3), tumor mutational burden (TMB) (4, 5), and gene expression profile (GEP) (6), have been recently associated with ICB response. However, the predictive values of these biomarkers are relatively limited because of the low predictive accuracies. Thus, the search for useful and precise biomarkers for predicting ICB response is critical.

Increasing studies have reported that mutated genes carrying single nucleotide variants (SNVs) are significantly related with the ICB response (7, 8). For example, *STK11*, *B2M*, and *EGFR* mutations or *MDM2* amplification were associated with poor responsiveness or even hyper-progressive disease (HPD) (9, 10), whereas *TP53*, *KRAS*, and *POLE* mutations or KP (co-mutations of *KRAS* and *TP53*) molecular sub-type were positively related with ICB response in advanced NSCLC (11, 12). Patients with KL (co-mutations of *KRAS* and *STK11*) showed poor responses (13, 14). Moreover, NSCLC patients with mutations or co-mutations of *DDR* and *Notch* pathways had clinical benefit from ICB (15–17). These findings reveal the potential values of exploiting a novel method for predicting clinical benefit using a mutational database.

Deep learning, especially as convolutional neural network (CNN), has frequently been applied to medical images for the diagnosis, predictive prognosis, and therapy response assessment of patients with cancer (18–20). However, there is unclear whether the CNN based on SNV database could predict the clinical outcome of immunotherapy in the patients with NSCLC.

Therefore, we sought to use CNN algorithm based on a panel of genomic mutations to develop a robust model for selecting patients with advanced NSCLC who are responsive to ICB therapy. CNN based on next-generation sequencing (NGS) and whole-exome sequencing (WES) databases was used to predict ICB benefit in patients with NSCLC from three large cohorts. The CNN model showed a better predictive ability than PD-L1, TMB, and conventional machine learning (cML) models. Moreover, we combined CNN and cML models to build a nomogram for predicting the prognosis of immunotherapy. A robust comprehensive classification of genomic panels would facilitate the selection of patients who would benefit from ICB.

## Materials and methods

### Patients treated with immunotherapy

*POPLAR/OAK cohort:* A total of 287 patients with advanced or metastatic NSCLC were recruited in the POPLAR study (NCT01903993) (21). The exclusion criteria were as follows: second or third-line standard therapy of docetaxel (n=143), treatment with atezolizumab (n=144), and no blood TMB (bTMB) data (n=39). Finally, 105 patients were retained and unchosen for PD-L1 expression status. The OAK study (NCT02008227), a randomized phase III trial, recruited 850 patients with metastatic NSCLC to compare atezolizumab with docetaxel in the primary analysis population (22). A total of 425 patients who received second or third-line standard therapy of docetaxel, 425 patients who were treated with atezolizumab, and 101 patients who had no bTMB data were excluded. Finally, 324 patients were retained and unchosen for PD-L1 expression status. The POPLAR and OAK cohorts had 429 patients in total as a training cohort.

*UCMC cohort:* Patients with locally advanced or metastatic NSCLC who were treated with only anti-PD-1 or a combination of chemo-immunotherapy treatment in the University of Chicago Medical Center (UCMC) were investigated (23). The patients had undergone tumor NGS test prior to the initiation of ICB therapy. Between 2016 and 2020, of the 426 patients treated with ICB, 139 who undergone tumor NGS test prior to the initiation of ICB therapy were deemed eligible to participate in this study. Two patients without TMB database were excluded. Finally, the tumor samples of 137 patients were evaluated for PD-L1 expression or EGFR/ALK mutational status, and results were validated using deep learning and cML algorithms.

*MSKCC cohort:* The Memorial Sloan Kettering Cancer Center (MSKCC) cohort of patients with advanced NSCLC receiving anti-PD-1 treatment were derived from three clinical studies. In the first cohort, 75 patients with stage IV NSCLC were treated with a combination of nivolumab and ipilimumab in the CheckMate-012 clinical trial (NCT01454102) between February 2013 and March 2015 (24). In the second cohort, 34 patients with metastatic NSCLC treated with anti-PD-1 treatment were derived from the MSKCC (n=29) and University of California at Los Angeles (n=5) (NCT01295827) studies (25). In the third cohort, the data of 240 patients treated with only anti-PD-1 or a combination of anti-CTLA-4 and anti-PD-1 between April 2011 and January 2017 were retrospectively collected (10). A total of 349 patients were considered as another validation cohort.

This study was approved by the institutional review board of the Second Affiliated Hospital of Guizhou Medical University and was conducted in accordance with the tenets of the Declaration of Helsinki.

### Study design

**Figure 1:**
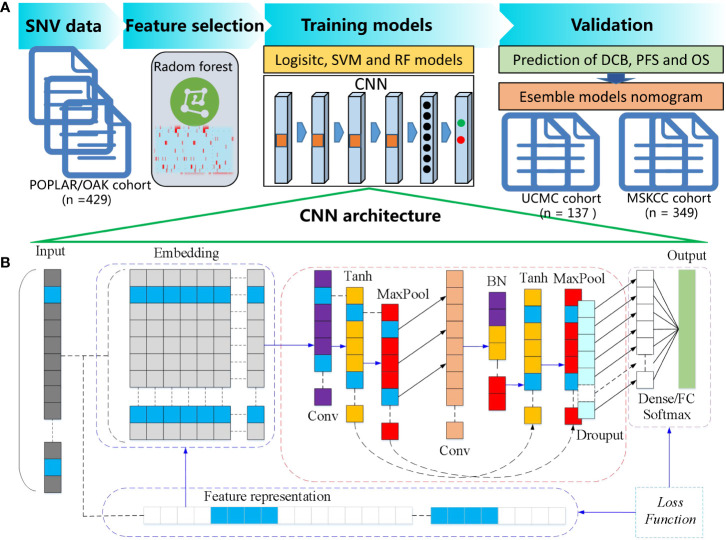
Flowchart of the proposed CNN and cML models for predicting DCB and prognosis. **(A)** SNV databases were collected from NSCLC patients before ICB treatment. In the POPLAR/OAK cohort, RF based on a five-fold cross-validation was used to select the optimal genomic features. Then, the selected genes were inputted into CNN, logistic, SVM, and RF models. The four machine learning models were validated for DCB in the UCMC and MSKCC cohorts. After multivariate analysis, the CNN and several cML models were combined to build a nomogram for predicting PFS and OS in the above mentioned three cohorts. **(B)** The detailed architecture of one-dimensional CNN is presented.

The flowchart of the proposed CNN and cML models for predicting DCB and prognosis is shown in **Figure 1A**. The SNV databases of sequencing results of tumor or blood samples from the patients with NSCLC before ICB treatment were collected. In the POPLAR/OAK cohort, the optimal genomic features were selected by RF algorithm based on a five-fold cross-validation as previously described (26). The selected genes were input into CNN, logistic, support vector machine (SVM), and random forest (RF) models and were used to train for DCB prediction. After adjusting the parameters, the four machine learning models were validated for DCB in the UCMC and MSKCC cohorts. The associations between the predictive scores of four models and prognosis were analyzed. CNN model was further trained on the data of the clinical subgroups. After multivariate analysis, the CNN and cML models were combined to build a nomogram for predicting PFS and OS in the above mentioned three cohorts.

### DCB, PFS, and OS

The primary outcome measures of this study were durable clinical benefit (DCB), progression-free survival (PFS), and overall survival (OS). The Response Evaluation Criteria in Solid Tumors (RECIST) version 1.1 were used to evaluate complete response (CR), partial response (PR), stable disease (SD), and progressive disease (PD). We defined DCB as CR, PR, or SD lasting ≥ 6 months, whereas no durable benefit (NDB) was defined as either SD lasting< 6 months or PD. We defined PFS as the time from the start of PD-1/PD-L1 blockade therapy to death or the first confirmation of PD based on RECIST version 1.1. OS was defined as the time from the start of PD-1/PD-L1 blockade therapy until the last contact or death.

### WES, targeted NGS, TMB, and PD-L1 analysis

WES and targeted NGS of tumor and blood samples were performed before ICB. DNA and circulating tumor DNA (ctDNA) were separately extracted from formalin-fixed paraffin- embedded (FFPE) tumor masses and peripheral blood samples from the patients. The different sequencing assays, including WES and targeted NGS (MSK-IMPACT and OncoPlus), were performed as described in **Supplementary Methods**.

Based on the results of WES and targeted NGS profiling, we defined a high TMB as ≥20/Mb or total somatic nonsynonymous as ≥ 200, and low TMB as<20/Mb or total nonsynonymous mutations as<200. The E1L3N (Cell Signaling, Danvers, MA, USA), 22C3 (DAKO), and 28-8 (DAKO) were used to determine the PD-L1 expression of tumor cells. Positive PD-L1 expression was defined as > 1% staining.

### Selection of genomics features and construction of conventional machine learning models

The genomics features of the SNVs were selected by RF function and five-fold cross-validation sampling. Three cML algorithms, including SVM, logistic, and RF, were employed. Based on the selected genomics features, three cML models were built by R packages (“randomForest”, “caret”, and “e1071”).

### CNN architecture

As shown in the **Figure 1B**, the architecture of the one-dimensional CNN included a one-dimensional convolution layer, with a convolution kernel of 16, a spatial domain of convolution kernel of 128, and a stride of 1. Firstly, the input information was processed as embedded. Secondly, we used the tanh activation function, followed by the Maxpooling method to reduce the dimension. After the first dimensionality reduction, one-dimensional convolution calculation was carried out for the vector, with a convolution kernel of 32 and a spatial domain of convolution kernel of 3. Then, batch normalization (BN) was carried out. Similarly, tanh activation function was used, and dimensionality was reduced by Maxpooling. Adam was used as deep neural network optimization, the gradient descent method was SGD, and the learning rate was 0.01. On the basis of the above, the fully-connected feed forward network (FCN) of dense and the output result of the Softmax activation function were used as previously described (27).


$$Z^{l+1}(i,j)=[Z^{l}⊗w^{l+1}](i,j)+b=\sum_{k=1}^{K_{i}}\sum_{x=1}^{f}\sum_{y=1}^{f}[Z_{k}^{l}(s_{0}i+x,s_{0}j+y)w_{k}^{l+1}(x,y)]+b$$

$$(i,j)\in \{0,1,\cdots ,L_{l+1}\}\;L_{l+1}=\frac{L_{l}+2p-f}{s_{0}}+1$$

The summation part in the above formula is equivalent to solving a cross correlation, where *b* is the deviation; *Z^l^* and *Z^l+^* ^1^ represent the convolution input and output of layers *l*+1, respectively, also known as feature map; *L_l+_*_1_ is the dimension of *Z_l_*_+1_; *K* is the number of channels; and *f*, *s*_0_, and *p* are the convolution layer parameters corresponding to the size of convolution kernel, convolution stride, and the number of padding layers, respectively.

The tanh activation function is shown below:


$$f\left(x\right)=\frac{e^{x}-e^{-x}}{e^{x}+e^{-x}}$$

The Softmax activation function is expressed as follows:


$$Si=\frac{e^{vi}}{\Sigma_{i}^{c}e^{vi}}$$

where *Vi* is the output of the output unit of the front stage of the classifier, *i* represents the category index, *C* is the total number of categories, and *Si* represents the ratio of the index of the current element to the sum of the indexes of all elements.

Cross Entropy Loss was calculated as below:


$$loss=-\left[y\text{log}\hat{y}+\left(1-y\right)\text{log}\left(1-\hat{y}\right)\right]$$

where *y* is the real value, and $\hat{y}$ is the predicted value.

### CNN implementation

The selected 55 genomics features were input to the CNN model. To ensure adequate performance of training process, the maximum number of epochs was set to 600. Implementation of deep learning was based on the TensorFlow-1.14 in Python (https://www.python.org/). The experiment was performed in a Windows environment with a 3.7 GHz Intel i7-12700KF CPU, NVIDIA GeForce RTX 3090, and 32 GB of RAM.

### Statistical analysis

The ROC curves were plotted and evaluated for accuracy using the “pROC” package. The area under the curve (AUC) and the corresponding 95% confidence interval (CI) were calculated in the three cohorts. PFS and OS curves were analyzed by Kaplan–Meier method and plotted by the survminer package. Multivariate Cox regression of the CNN, logistic, SVM, and RF models was analyzed, and significant variables (*P*< 0.01) were used to build the nomogram using “rms” package. The HRs for PFS and OS in the CNN-low and CNN-high subgroups were analyzed and visualized by the “Forestplot” package. KEGG (Kyoto Encyclopedia of Genes and Genomes) and Gene Ontology (GO) were analyzed in DAVID (https://david.ncifcrf.gov/home.jsp). All statistical analyses were conducted in R version 3.5.1 (https://www.r-project.org/) and GraphPad Prism 7.01 (https://www.graphpad.com/). *P*< 0.05 was defined as statistically significant.

## Results

### Characteristics of patients who received ICB therapy

The basic clinical characteristics of the patients with NSCLC in the POPLAR/OAK, UCMC, and MSKCC cohorts are presented in **Supplementary Table 1**. A total of 275 (64.10%), 61 (44.53%), and 171 (49.00%) patients were men in the POPLAR/OAK, UCMC, and MSKCC cohorts, respectively. In the three cohorts, there were 265 (61.77%), 92 (67.15%), and 222 (63.61%) patients aged >60 years old. Majority (82.05%, 87.59%, and 80.51%) of the patients were current or ever smokers. Patients with no-squamous NSCLC comprised 70.86% and 94.26% of the POPLAR/OAK and MSKCC cohorts. We found that 15 (3.50%), 20 (14.60%), and 71 (20.34%) patients had high TMB (≥200 or >20/Mb) in the three cohorts and that the TMB status is a stratifying variable in the different populations. In the POPLAR/OAK, UCMC, and MSKCC cohorts, 59 (13.75%), 45 (32.84%), and 43(12.33%) patients had positive PD-L1 expression (>1%), respectively. There were 286 (31.27%) patients in the three cohorts who were not tested for PD-L1 expression. In the POPLAR/OAK, UCMC, and MSKCC cohorts, 134 (31.24%), 57 (41.61%), and 131 (37.54%) patients achieved DCB, respectively.

### Landscape of selection genomics and building of cML for DCB

**Figure 2:**
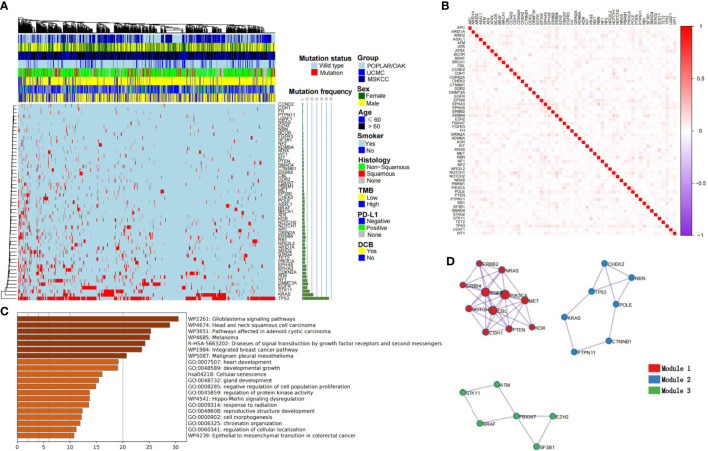
Summary characteristics of somatic mutations and construction of cML models. **(A)** The clinical factors and frequency of the 55 selected genes are presented in the heatmap. **(B)** The relationships among each genomic feature are shown. **(C)** The KEGG, GO analyses of the 55 mutational genes. **(D)** Three clusters are presented as module1, module 2 and module 3.

Based on the five-fold cross-validation, the RF was used to select the optimal mutational genomics from the POPLAR/OAK cohort, and 55 somatic mutations were finally chosen. The importance and Gini coefficients of the top 30 somatic mutations are shown in **Supplementary Figure 1**. The ARID1A and ERBB4 showed the important roles of the 55 genomics features. We summarized the clinical and somatic mutations in the three patient cohorts with NSCLC (**Figure 2A**). Seven mutational subtypes (nonsense mutation, missense mutation, frame shift del, frame shift ins, splice site, inframe del, and multi hit) were detected in the three cohorts, and the frequencies of the 55 selected genes in each case are shown as a heatmap. *TP53*, *KRAS*, and *STK11* showed high mutational frequency (55.30%, 22.62%, and 15.19%) in the total cohort (n = 915). The correlations among 55 somatic mutations are presented in **Figure 2B**. *Notch1* and *POLE* had a positive correlation (r = 0.133, *P<* 0.001), whereas *KRAS* and *EGFR* showed a negative correlation (r = −0.150, *P<* 0.001).

KEGG revealed 55 mutational genes that are associated with different cancer pathways, such as the glioblastoma signaling pathway, head and neck squamous cell carcinoma, and melanoma (FDR *P<* 0.001; **Figure 2C**). GO analysis showed that theses genomics are related with development growth, regulation of protein kinase activity (PKA), and response to radiation. Modular analysis revealed 55 genes that could be classified into three groups. Model1 consisted of *ERBB2*, *ERBB4*, *NRAS*, *EGFR*, *PIK3CA*, *NOTCH1*, *CBL*, *MET*, *CDH1*, *PTEN*, and *KDR* (**Figure 2D**). Based on the three cML algorithms, we used the panel of 55 mutational genes to train the model to predict DCB. The training process of RF is shown in **Supplementary Figure 2**, with the number of trees set at 100. The RF model showed a better and more stable prediction than the logistic and SVM methods in the POPLAR/OAK, UCMC, and MSKCC cohorts (**Supplementary Table 2**).

### Convolutional neural network was trained and tested for DCB in the three ICB cohorts

**Figure 3:**
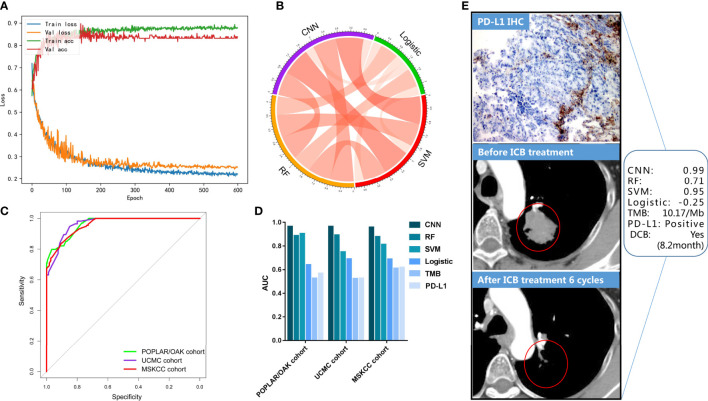
CNN training and validation for DCB in the three cohorts. **(A)** The accuracy and loss are plotted for the training and validation processes. **(B)** The correlations of predictive scores among CNN, logistic, SVM, and RF models. **(C)** The ROCs were plotted in the three cohorts. **(D)** AUCs of the PD-L1, TMB, logistic, SVM, and CNN models are shown. **(E)** A representative NSCLC case treated with ICB is shown, and the evaluation of the PD-L1, TMB, logistic, SVM, and CNN models are presented.

The deep-learning model of CNN was implemented in the TensorFlow platform as the set parameters. The POPLAR/OAK cohort was trained, and the UCMC cohort was validated in the process of 600 epochs (**Figure 3A**). The curves of training accuracy and loss had consistent trend with the validating curves. Then, the MSKCC cohort was tested using the trained CNN model. We found significant associations between the three cML (logistic, SVM, and RF) models and the CNN model (each *P<* 0.001) (**Figure 3B**), with the strongest correlation between the CNN and SVM models (r = 0.905, 95% CI: 0.886–0.920, *P*< 0.001). The CNN model had the highest AUCs in the POPLAR/OAK, UCMC, and MSKCC cohorts (AUC = 0.965, 95% CI: 0.949–0.978, *P<* 0.001; AUC =0.965, 95% CI: 0.940–0.989, *P*< 0.001; AUC = 0.959, 95% CI: 0.942–0.976, *P*< 0.001, respectively; **Figure 3C**). Comparing the predictive accuracy of TMB, PD-L1, logistic, SVM, RF, and CNN, the CNN had the highest AUCs in the three cohorts, and we found that TMB and PD-L1 had similar AUCs (**Figure 3D**). The CNN model also had significantly higher sensitivity and specificity than TMB and PD-L1 in the POPLAR/OAK cohort (sensitivity = 97.01, 95% CI: 92.53–99.18, *P<* 0.001; specificity = 76.95, 95% CI: 71.72–81.63, *P*< 0.001), the UCMC cohort (sensitivity = 94.74, 95% CI: 85.38–98.90, *P<* 0.001; specificity = 85.00, 95% CI: 75.26–92.00, *P*< 0.001), and the MSKCC cohort (sensitivity = 83.97, 95% CI: 76.55–89.79, *P<* 0.001; specificity = 90.37, 95% CI: 85.65–93.94, *P*< 0.001; **Supplementary Table S3**). A case with a positive PD-L1 expression and a low TMB showed a significant response. The somatic mutations included *TP53*, *KRAS*, *EPHA5*, *ARID1A*, and *POLE*. The CNN and the three cML models showed different scores in this patient (**Figure 3E**).

### Convolutional neural network and conventional machine learning predict PFS and OS in NSCLC patients with ICB

**Figure 4:**
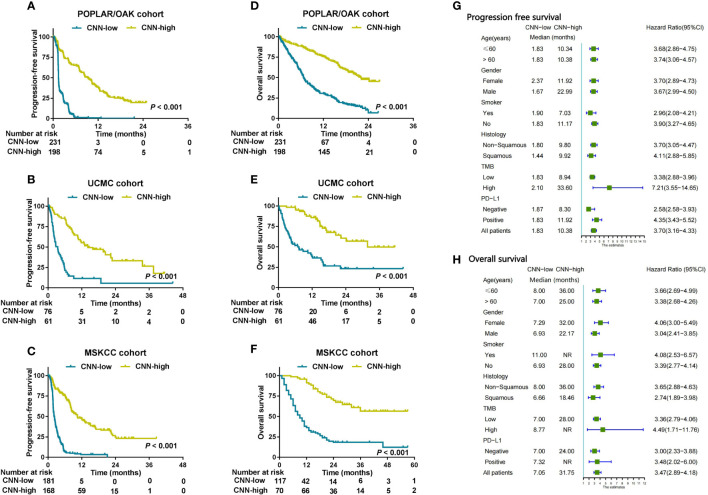
CNN was used to predict prognosis and analyzed in clinical subgroups. **(A–C)** Kaplan–Meier survival curves showing PFS between the CNN-low and CNN-high groups in patients from the three cohorts. **(D–F)** Kaplan–Meier survival curves showing OS between the CNN-low and CNN-high groups in three cohorts. **(G, H)** Subgroup analysis of CNN for PFS and OS from the combination cohorts (n = 915) according to basic clinical variables.

According to the cut-off value (0.31) of CNN scores as the biggest Youden Index (YI), the patients with NSCLC treated with ICB were stratified into CNN-high (> 0.31) or CNN-low (≤ 0.31) groups. The CNN-high group had a longer median PFS than that of the CNN-low group (mPFS: 1.41 vs 9.29 months) (HR = 3.67 [2.94–.57], *P*< 0.001; **Figure 4A**) in the POPLAR/ OAK cohort with anti-PD-1 therapy. In the UCMC and MSKCC cohorts, the CNN-high group also showed better PFS than that by the CNN-low group (both *P*< 0.001; **Figures 4B, C**). We then validated the predictive OS of the CNN model in the POPLAR/OAK cohort and found that the CNN-high group had a longer median OS (mOS: 6.70 vs 22.04 months) (HR = 3.20 [2.52–4.06], *P*< 0.001) than that of the CNN-low group (**Figure 4D**). The CNN-high group had better OS than that of the CNN-low group in both the UCMC and MSKCC cohorts (both *P*< 0.001; **Figures 4E, F**). PD-L1 expression was a significant predictor of PFS in the MSKCC cohort (*P<* 0.001), and TMB was a significant biomarker for PFS in the POPLAR/OAK and MSKCC cohorts (*P* = 0.004 and *P*< 0.001; **Supplementary Table 4**). PD-L1 expression was a significant predictor of OS in the POPLAR/OAK and MSKCC cohorts (*P* = 0.005 and *P* = 0.022), and TMB was a significant predictor of OS in the MSKCC cohort (*P* = 0.049, **Supplementary Table 5**). The three cML models accurately predicted the OS and PFS in the three cohorts (**Supplementary Tables 4, 5**). We analyzed the prediction of CNN in the clinical subgroups across different variables. Combining the three cohorts, the CNN model showed a good prediction as a biomarker of PFS and OS (**Figures 4G–H**).

### Nomogram of combining CNN, SVM, and RF predicts PFS and OS in patients with ICB

In the POPLAR/OAK cohort, we used six variables (including logistic, SVM, RF, CNN, TMB, and PD-L1) for the multivariate analysis of PFS and OS. The SVM, RF, and CNN models were identified as significant independent factors for PFS (*P<* 0.001, 0.014, and< 0.001) and SVM, CNN, and PD-L1 (*P<* 0.001,< 0.001, and 0.005) were significant independent factors for OS (**Supplementary Table 6**).

**Figure 5:**
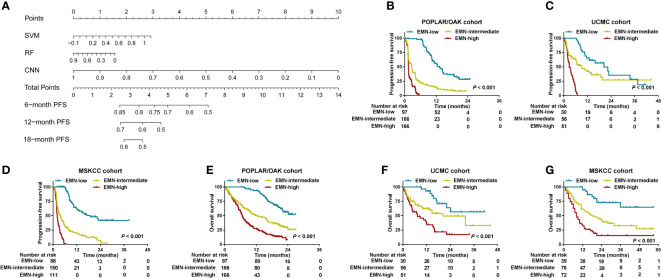
Development and analysis of a nomogram for PFS and OS in the three cohorts. **(A)** A nomogram was constructed using SVM, RF, and CNN methods in the POPLAR/OAK cohort. **(B–D)** PFS curves were compared among the EMN-low, EMN-intermediate, and EMN-high subgroups of patients from the three cohorts. **(E–G)** OS curves were compared among the EMN-low, EMN-intermediate, and EMN-high subgroups of patients from the three cohorts.

Considering the low predictive ability of PD-L1 expression for prognosis, we used the SVM, RF, and CNN models to develop a comprehensive nomogram for predicting PFS (herein, EMN; **Figure 5A**). According to the two cut-off values (0 and 0.8), we stratified the three cohorts into EMN-low (≤0), EMN-intermediate (0< and ≤ 0.8), and EMN-high (> 0.8) groups. The EMN-low had a longer median PFS and OS than the EMN-intermediate and EMN-high groups (mPFS: 13.37 vs 2.72 vs 1.41 months, *P*< 0.00; mOS: not reached [NR] vs 12.41 vs 6.66 months, *P*< 0.001) (**Figures 5B, E**) in the POPLAR/OAK cohort. Similarly, the patients with EMN-low had better PFS and OS than those with EMN-intermediate and EMN-high in the UCMC and MSKCC cohorts (mPFS: 20.41 vs 8.94 vs 2.79 months, *P*< 0.001; 18.90 vs 3.60 vs 2.10 months, *P*< 0.001; mOS: NR vs 20.42 vs 7.36 months, *P*< 0.001; NR vs 18.00 vs 8.00 months, *P<* 0.001; **Figures 5C–E, G**).

## Discussion

In this study, we used deep learning and cML methods based on the NGS or WES data to develop predictive models for DCB in 915 patients with NSCLC treated with ICB from three independent cohorts. To our knowledge, this is the largest study conducted to predict ICB response based on the sequencing data of patients with NSCLC. To avoid overfitting of the training model, the RF algorithm was first used to reduce the genomics features, and 55 somatic mutations were finally selected. After tuning the parameters, the CNN and cML models showed high prediction accuracies for DCB in the POPLAR/OAK, UCMC, and MSKCC cohorts. In the four models, namely CNN, SVM, RF, and logistic, a significant association with PFS and OS in the above three cohorts was noted. Subgroup analysis of CNN revealed that deep learning had robust prediction in different clinical variables. After multivariate analysis, we used SVM, RF, and CNN to build a nomogram for predicting PFS and found that this nomogram could stratify patients into three groups. The three groups of patients had different PFS and OS in the POPLAR/OAK, UCMC, and MSKCC cohorts.

Although WES, WGS, and NGS databases from blood or tumor tissue samples have been increasingly and extensively used in cancer research, most studies have frequently focused on several gene panels or sole driver mutational gene, resulting in the inefficient use of large sequencing data, especially in somatic mutations (28–30). In this study, we mainly sought to use a relatively small panel of mutational genes to develop a robust predicting model for ICB response. Comparing the different cML models (SVM, RF, and logistic), we found that the CNN model had the highest precision for DCB prediction. This was the first study that used CNN to train somatic mutations but not routine images. More research should be conducted to determine whether CNN can potentially perform on classification of predicting DCB from large WES, NGS, or WGS data but not use a simple TMB. We also found CNN had a higher and more stable ability for predicting DCB than TMB and PD-L1 expression status. Because our genomics sequencing data were collected from circulating tumor DNA (ctDNA) and analysis in the training model (POPLAR/OAK) were validated from the other two cohorts (UCMC and MSKCC) with tumor tissue sequencing, these findings reveal that our CNN model could be potentially used as a non-invasive method to predict ICB response of patients with NSCLC.

Previous studies have reported that patients with various cancers who are responsive to ICB treatment frequently have a better prognosis than nonresponsive patients (31, 32). The CNN, SVM, logistic, and RF models for predicting DCB were significantly associated with PFS and OS. The patients with CNN-high had significantly better PFS and OS than those with CNN-low in the POPLAR/OAK, UCMC, and MSKCC cohorts. This finding revealed that the CNN model for predicting DCB could effectively assess the clinical prognosis of ICB therapy in patients with NSCLC. In contrast, TMB and PD-L1 expression showed unsatisfactory results for predicting PFS and OS in the three cohorts. We speculated that the different cut-off values or detection platform for TMB could have contributed to the uncertain predictive effect. New method of tumor mutational burden on cytological samples from a pilot study could be feasible and the application was unclear (33). PD-L1 is becoming a fundamental data for patient management and the role of pathologist and immunohistochemical assessement should be emphasized (34). The testing results of the PD-L1 assay varied across various reagents from several manufacturers (35). New evidences regarding clones, platforms, reporting system are issues (36). Additionally, PD-L1 expression from different areas of the tumor may also differ (37). Therefore, using PD-L1 as a prognostic and predictive marker of response to therapy was insufficient. This suggests that our CNN model is a feasible tool and could supplement the limitations of PD-L1 expression and TMB for predicting the PFS and OS in patients with NSCLC who were treated with ICB.

In the combination of three cohorts, subgroup analysis of patients with CNN-high also had significantly longer PFS and OS than those with CNN-low. These results support that the CNN model had a good prognostic prediction and was not affected by clinical variables. Combining deep learning and cML methods based on radiology images was suggested in previous studies and using this combination could improve the predictive abilities of various models (38–40). Interestingly, this is the first study to integrate CNN and cML methods based on WES and NGS databases to construct a nomogram for the stratification of patients treated with ICB. We found that patients in the EMN-low, EMN-intermediate, and EMN-high groups had different PFS and OS. This finding indicated that ensemble models, such as CNN, SVM, and RF, are promising and precise tools for predicting clinical benefit (for example, DCB, PFS, and OS) in patients undergoing immunotherapy.

However, this study had some limitations. First, although the number of patients was relatively large, the POPLAR/OAK, UCMC, and MSKCC cohorts were obtained from the American population. Thus, the molecular characteristics of these NSCLC patients may differ from those of East Asian descent, thus affecting clinical treatment outcomes (41). The CNN model should be trained with large international multi-center datasets to further improve prediction performance. Second, a panel of genomic variants based on the WES or NGS data was used in this study; however, DNA methylation, mRNA expression, radiology, and pathology were not used to predict the DCB. A multi-omics model, in addition to a genomics model, should be studied. Third, although extraction of the ctDNA of peripheral blood samples is a non-invasive method, the predictive ability of the model requires further investigation.

In summary, CNN classification based on a panel of 55 mutational genes serves as a novel and robust model for predicting DCB from ICB therapy in patients with NSCLC. A combination model that integrated CNN, SVM, and RF algorithms could better predict the PFS and OS. These findings may contribute to the discovery of a new strategy for patients with NSCLC treated with PD-1/PD-L1 blockade. Our method based on NGS and WES databases provides new insights for predicting clinical outcome in pan-cancer immunotherapy.

## Data availability statement

The raw data supporting the conclusions of this article will be made available by the authors, without undue reservation. The data has been resubmitted to Dryad (https://doi.org/10.5061/dryad.hdr7sqvmf).

## Author contributions

(I) Conception and design: JP; (II) Administrative support: JP; (III) Provision of study materials or patients: JP and DZ; (IV) Collection and assembly of data: JP; (V) Data analysis and interpretation: JP; (VI) Manuscript writing: All authors; (VII) Final approval of manuscript: All authors.

## Funding

This work was supported by National Nature Science Foundation of China (grant number: 82060327); the Science and Technology Foundation of Guizhou Province (grant numbers: Qian ke he ji chu-ZK 2021 and yi ban 454); and the Qian Dong Nan Science and Technology Program (grant number: qdnkhJz2020-013).

## Conflict of interest

Author BX is employed by Yino Research.

The remaining authors declare that the research was conducted in the absence of any commercial or financial relationships that could be construed as a potential conflict of interest.

## Publisher’s note

All claims expressed in this article are solely those of the authors and do not necessarily represent those of their affiliated organizations, or those of the publisher, the editors and the reviewers. Any product that may be evaluated in this article, or claim that may be made by its manufacturer, is not guaranteed or endorsed by the publisher.
