# Supplementary material for: Deep learning to estimate durable clinical benefit and prognosis from patients with non-small cell lung cancer treated with PD-1/PD-L1 blockade
Source: Front Immunol. 2022 Nov 7;13:960459. doi: 10.3389/fimmu.2022.960459 (PMC9677530; doi:10.3389/fimmu.2022.960459)
Supplement: Supplementary file 1 [file DataSheet_1.docx]

Supplementary Material

# Supplementary Methods

**Sequencing methods**

1. **WES**: The whole exome was captured using the Illumina Rapid Capture Exome Target Bait Kit (38 MB) and Agilent Sure-Select Human All Exon v2.0 (44 MB)/v4.0 (51 MB) according to the manufacturer’s instructions. The HiSeq 2000, 2500, or 4000 platform (Illumina, San Diego, California) was used to provide 150× the average target coverage, extensive exon library, and paired reads (2×76 bp). For each sample, the Burrows–Wheeler Aligner was used to generate a normal BAM file, and the tumour sequence was compared to the human hg19 genome construct. The Genome Analysis Toolkit (GATK) was used to analyse basic quality factor recalibration, indel recombination, and duplicate deletion. The MuTect v1.1 .6 software (Broad Institute Picard) was used to calculate quality assurance indicators based on predefined parameters that were instrumental to generate single nucleotide variation (SNV) calls. Indel calls were generated using the Indelocator software (http://archive.broadinstitute. org/). Mutations with an allelic fragment variation; 0.05 were excluded. Location-based artifact filtering was used for variant allelic mutations from independent groups of normal exons from blood samples of non-cancer patients. Ensemble edition 75 was used to describe the PyEnsembl and Varcode variants. The TMB in each sample was defined as the total number of non-synonymous mutations, including SNVs and indels.
2. **Targeted NGS (MSK-IMPACT)**: After generation and sequencing, the barcode library targeted exons and chose introns of 468, 410, or 341 genes. In all tumour samples, the average sequence index was 7443, and the minimum coverage depth was 913. A custom pipeline was used to identify the somatic alterations in the tumour samples. To normalise somatic non-synonymous TMB on panels of different sizes, we divided the detected coding regions in each panel by the total number of mutations, which covers 0.98 Mb, 1.06 Mb, and 1.22 Mb of the 341, 410, and 468 gene panels, respectively.
3. **Targeted NGS (OncoPlus:)**: At the UCMC Clinical Genomic and Molecular Pathology Laboratory, 1212 genes were analysed by targeted NGS. Sequencing reads were aligned to the human genome (hg19) by Burrows–Wheeler Alignment version 0.7 .241. The ABRA version 0.9642 was used to improve the detection of complex genomic variants. Based on the Picard Mark Duplicates version 1.92 or 2.81 (Broad Institute), duplicate reads introduced during amplification were removed. The Alamut Interactive BioSoftware version 1.4 .4 or 1.11 was used to annotate variants. Variants with a variant allele fraction (VAF) of >5% were filtered out. Variants were interpreted according to the 2017 AMP, ASCO, and CAP standards.

# Supplementary Figures and Tables

## Supplementary Figures


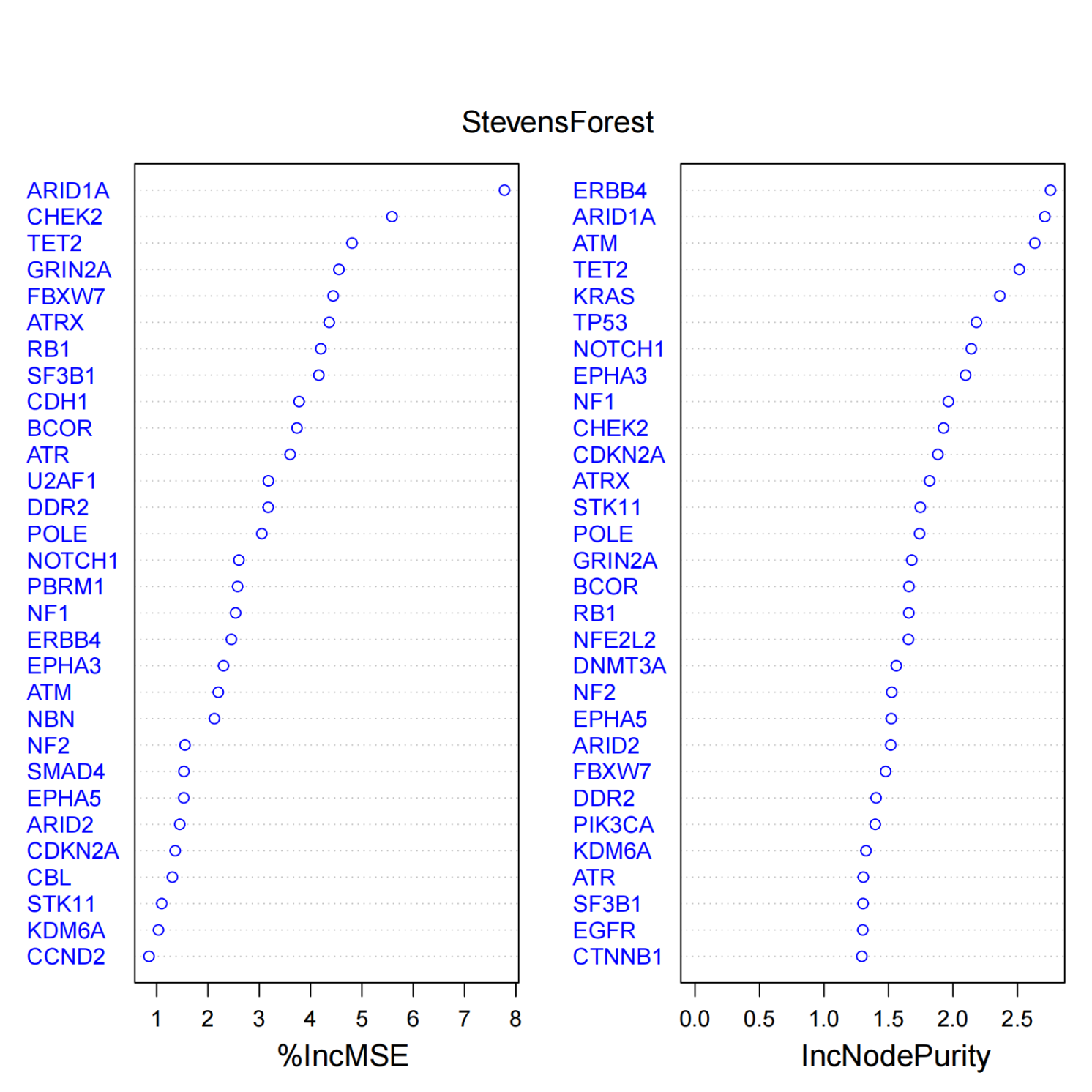


**Supplementary Figure 1.** The Gini coefficients and IncNodePurity of top 30 somatic mutations were presented in RF method of selecting features.


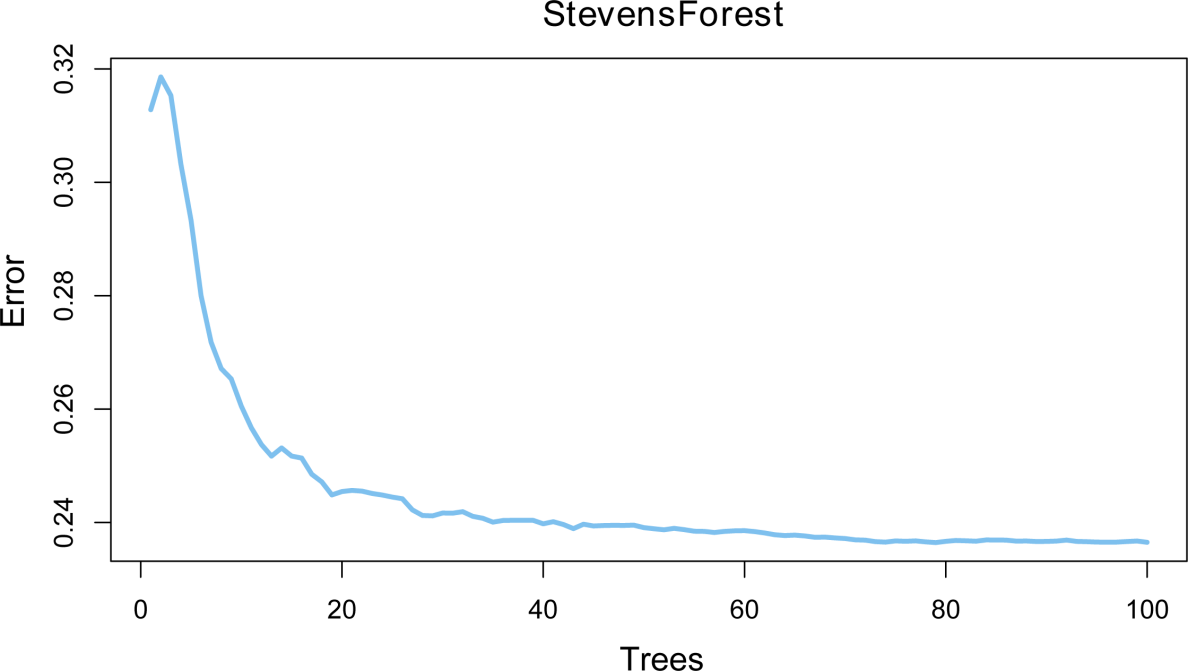


**Supplementary Figure 2.** The training process of RF model in the POPLAR/OAK cohort is shown, with 100 set of trees.

## Supplementary Tables

**Supplementary Table 1. Characteristics of patients in the three cohorts**

| **Variable** | **Total**  **(N = 915)** | **POPLAR/OAK cohort (n = 429)** | **UCMC cohort**  **(n = 137)** | **MSKCC cohort**  **(n = 349)** |
| --- | --- | --- | --- | --- |
| Sex |  |  |  |  |
| Female | 408 (44.59%) | 154 (35.90%) | 76 (55.47%) | 178 (51.00%) |
| Male | 507 (55.41%) | 275 (64.10%) | 61 (44.53%) | 171 (49.00%) |
| Age (years) |  |  |  |  |
| ≤ 60 | 336 (36.73%) | 164 (38.23%) | 45 (32.85%) | 127 (36.39%) |
| > 60 | 579 (63.27%) | 265 (61.77%) | 92 (67.15%) | 222 (63.61%) |
| Smoker |  |  |  |  |
| Yes | 758 (82.84%) | 352 (82.05%) | 120 (87.59%) | 281 (80.51%) |
| No | 162 (17.16%) | 77 (17.95%) | 17 (12.41%) | 68 (19.49%) |
| Histology |  |  |  |  |
| Non-squamous | 633 (81.36%) | 304 (70.86%) | NA | 329 (94.26%) |
| Squamous | 145 (18.64%) | 125 (29.14%) | NA | 20 (5.74%) |
| TMB |  |  |  |  |
| Low | 809 (88.41%) | 414 (96.50%) | 117 (85.40%) | 278 (79.66%) |
| High | 106 (11.59%) | 15 (3.50%) | 20 (14.60%) | 71 (20.34%) |
| PD-L1 |  |  |  |  |
| Negative | 482 (52.67%) | 263 (61.30%) | 67 (48.90%) | 152 (43.55%) |
| Positive | 147 (16.06%) | 59 (13.75%) | 45 (32.84%) | 43 (12.33%) |
| NA | 286 (31.27%) | 107 (24.95%) | 25 (18.26%) | 154 (44.12%) |
| DCB |  |  |  |  |
| Yes | 322 (35.19%) | 134 (31.24%) | 57 (41.61%) | 131 (37.54%) |
| No | 593 (64.81%) | 295 (68.76%) | 80 (58.39%) | 218 (62.46%) |
| Data are presented as n (%).  NA, not available. | | | | |

**Supplementary Table 2. Performance of machine learning in treatment response prediction.**

| **Groups** | **AUC (95% CI)** | **Sensitivity (95% CI)** | **Specificity (95% CI)** | ***P* value** |
| --- | --- | --- | --- | --- |
| **POPLAR/OAK cohort** |  |  |  |  |
| Logistic | 0.641 (0.584–0.697) | 64.93 (56.21–72.96) | 57.63 (51.77– 63.33) | < 0.001* |
| SVM | 0.906 (0.869–0.942) | 82.09 (74.53–88.17) | 87.12 (82.75–90.72) | < 0.001* |
| RF | 0.887 (0.853–0.921) | 80.60 (72.88–86.92) | 75.93 (70.64– 80.7) | < 0.001* |
| **UCMC cohort** |  |  |  |  |
| Logistic | 0.691 (0.604–0.778) | 70.18 (56.60–81.57) | 53.57 (42.24–64.97) | < 0.001* |
| SVM | 0.751 (0.657–0.846) | 70.18 (56.60–81.57) | 68.75 (57.41– 78.65) | < 0.001* |
| RF | 0.893 (0.841–0.945) | 80.70 (68.09–89.95) | 83.75 (73.82–91.05) | < 0.001* |
| **MSKCC cohort** |  |  |  |  |
| Logistic | 0.690 (0.632–0.748) | 60.31 (51.39–68.74) | 67.89 (61.25–74.04) | < 0.001* |
| SVM | 0.813 (0.757–0.870) | 76.34 (68.12–83.32) | 69.27 (62.68–75.32) | < 0.001* |
| RF | 0.879 (0.842–0.914) | 74.81 (66.48–81.98) | 80.73 (74.86–85.75) | < 0.001* |
| **P* value < 0.05. | | | | |

**Supplementary Table 3. Performance of TMB, PD-L1, and CNN in treatment response prediction.**

| **Groups** | **AUC (95% CI)** | **Sensitivity (95% CI)** | **Specificity (95% CI)** | ***P* value** |
| --- | --- | --- | --- | --- |
| **OAK/PLOAK cohort** |  |  |  |  |
| TMB | 0.528 (0.468–0.588) | 7.46 (3.63–13.30) | 98.31 (96.09–99.45) | 0.338 |
| PD–L1 | 0.570 (0.500–0.639) | 28.00 (19.48–37.87) | 86.04 (80.77–90.31) | 0.043* |
| CNN | 0.965 (0.949–0.978) | 97.01 (92.53–99.18) | 76.95 (71.72–81.63) | < 0.001* |
| **UCSC cohort** |  |  |  |  |
| TMB | 0.525 (0.426–0.624) | 17.54 (8.747– 29.91) | 87.50 (78.21– 93.84) | 0.616 |
| PD–L1 | 0.528 (0.418–0.637) | 43.48 (28.93–58.89) | 62.12 (49.34–73.78) | 0.615 |
| CNN | 0.965 (0.940–0.989) | 94.74 (85.38–98.90) | 85.00 (75.26–92.00) | < 0.001* |
| **MSKCC cohort** |  |  |  |  |
| TMB | 0.612 (0.549–0.675) | 34.35 (26.28–43.15) | 88.07 (83.01–92.06) | < 0.001* |
| PD–L1 | 0.619 (0.533–0.696) | 35.37 (25.12–46.70) | 87.61 (80.09–93.06) | < 0.001* |
| CNN | 0.959 (0.942–0.976) | 83.97 (76.55–89.79) | 90.37 (85.65–93.94) | < 0.001* |
| **P* < 0.05. | | | | |

**Supplementary Table 4. Performance of cML and CNN in PFS prediction.**

| **Groups** | **HR (95% CI)** | **mPFS (CNN-low)** | **mPFS (CNN-high)** | ***P* value** |
| --- | --- | --- | --- | --- |
| **POPLAR/OAK cohort** |  |  |  |  |
| Logistic | 1.40 (1.15–1.72) | 1.93 | 4.12 | < 0.001* |
| SVM | 2.61 (2.07–3.29) | 1.41 | 5.55 | < 0.001* |
| RF | 2.58 (2.10–3.17) | 1.51 | 8.27 | < 0.001* |
| CNN | 3.67 (2.94–4.57) | 1.41 | 9.29 | < 0.001* |
| TMB | 2.49 (1.61–3.84) | 2.69 | 12.45 | 0.004* |
| PD-L1 | 1.30 (0.99–1.72) | 2.56 | 4.46 | 0.076 |
| **UCMC cohort** |  |  |  |  |
| Logistic | 1.56 (1.03–2.37) | 5.72 | 10.62 | 0.038* |
| SVM | 1.80 (1.16–2.79) | 3.91 | 9.24 | 0.004* |
| RF | 2.75 (1.81–4.20) | 3.58 | 14.17 | < 0.001* |
| CNN | 3.36 (2.14–5.25) | 3.45 | 14.17 | < 0.001* |
| TMB | 1.39 (0.81–2.39) | 6.05 | 10.55 | 0.278 |
| PD-L1 | 1.25 (0.78–2.00) | 6.87 | 8.48 | 0.345 |
| **MSKCC cohort** |  |  |  |  |
| Logistic | 1.65 (1.29–2.12) | 3.07 | 6.13 | < 0.001* |
| SVM | 2.16 (1.66–2.83) | 2.52 | 6.33 | < 0.001* |
| RF | 2.65 (2.08–3.39) | 2.57 | 8.30 | < 0.001* |
| CNN | 4.00 (3.08–5.19) | 2.20 | 10.43 | < 0.001* |
| TMB | 2.67 (2.01–3.40) | 3.30 | 10.40 | < 0.001* |
| PD-L1 | 2.32 (1.62–3.34) | 4.10 | 14.50 | < 0.001* |
| **P* value < 0.05. | | | | |

**Supplementary Table 5. Performance of cML and CNN in OS prediction.**

| **Groups** | **HR (95% CI)** | **mOS (low score)** | **mOS (high score)** | ***P* value** |
| --- | --- | --- | --- | --- |
| **POPLAR/OAK cohort** |  |  |  |  |
| Logistic | 1.37 (1.09–1.73) | 10.28 | 15.90 | 0.006* |
| SVM | 2.39 (1.86–3.06) | 7.75 | 18.39 | < 0.001* |
| RF | 1.95 (1.54–2.46) | 8.31 | 19.94 | < 0.001* |
| CNN | 3.20 (2.52–4.06) | 6.70 | 22.04 | < 0.001* |
| TMB | 1.90 (1.09–3.32) | 12.41 | NR | 0.085 |
| PD-L1 | 1.72 (1.24–2.38) | 11.07 | 22.17 | 0.005* |
| **UCMC cohort** |  |  |  |  |
| Logistic | 1.54 (0.96–2.49) | 15.15 | 24.18 | 0.085 |
| SVM | 1.58 (0.97–2.57) | 12.13 | 22.05 | 0.052 |
| RF | 3.16 (1.97–5.07) | 8.77 | 31.75 | < 0.001* |
| CNN | 3.73 (2.30–6.04) | 6.90 | 31.75 | < 0.001* |
| TMB | 1.97 (1.03–3.76) | 15.61 | NR | 0.104 |
| PD-L1 | 0.92 (0.53–1.58) | 15.94 | 20.42 | 0.769 |
| **MSKCC cohort** |  |  |  |  |
| Logistic | 1.49 (1.03–2.17) | 11.00 | 22.00 | 0.029* |
| SVM | 1.40 (0.94–2.08) | 12.00 | 19.00 | 0.069 |
| RF | 2.12 (1.46–3.07) | 11.00 | 32.00 | < 0.001* |
| CNN | 4.49 (3.10–6.51) | 9.00 | NR | < 0.001* |
| TMB | 2.08 (1.18–3.67) | 15.00 | NR | 0.049* |
| PD-L1 | 2.80 (1.39–5.64) | 21.00 | NR | 0.022* |
| **P* < 0.05. | | | | |

**Supplementary Table 6. Multivariate analysis of PFS and OS in the POPLAR/OAK cohort.**

| **Groups** | **HR (95% CI)** | ***P* value** |
| --- | --- | --- |
| **PFS** |  |  |
| Logistic | 0.94 (0.73–1.20) | 0.642 |
| SVM | 1.68 (1.27–2.22) | < 0.001* |
| RF | 1.45 (1.07–1.97) | 0.014* |
| CNN | 3.50 (2.50–4.88) | < 0.001* |
| TMB | 1.42 (0.62–3.25) | 0.403 |
| PD-L1 | 1.26 (0.93–1.27) | 0.128 |
| **OS** |  |  |
| Logistic | 0.89 (0.66–1.20) | 0.470 |
| SVM | 1.74 (1.26–2.42) | < 0.001* |
| RF | 1.08 (0.78–1.50) | 0.636 |
| CNN | 2.34 (1.64–3.34) | < 0.001* |
| TMB | 1.12 (0.45–2.80) | 0.794 |
| PD-L1 | 1.73 (1.17–2.56) | 0.005* |
| **P* value < 0.05. | | |
